# Usefulness of discharge standards in outpatients undergoing sedative endoscopy: a propensity score-matched study of the modified post-anesthetic discharge scoring system and the modified Aldrete score

**DOI:** 10.1186/s12876-022-02549-7

**Published:** 2022-11-04

**Authors:** Daisuke Yamaguchi, Tomohito Morisaki, Yasuhisa Sakata, Yumi Mizuta, Goshi Nagatsuma, Suma Inoue, Akane Shimakura, Amane Jubashi, Yuki Takeuchi, Kei Ikeda, Yuichiro Tanaka, Wataru Yoshioka, Naoyuki Hino, Keisuke Ario, Seiji Tsunada, Motohiro Esaki

**Affiliations:** 1 Department of Gastroenterology, National Hospital Organization Ureshino Medical Center, Ureshino, Japan; 2 Division of Gastroenterology, Department of Internal Medicine, Saga University, Saga, 849-8501 Japan; 3 Department of Gastroenterology, Japan Community Health care Organization Isahaya General Hospital, Nagasaki, Japan

**Keywords:** Discharge, Sedation, Gastrointestinal endoscopy, Outpatients, Discharge time, Adverse events

## Abstract

**Background:**

This study aimed to evaluate the usefulness of discharge standards in outpatients undergoing sedative endoscopy by comparing the modified post-anesthetic discharge scoring system (MPADSS) and the modified Aldrete score.

**Methods:**

We prospectively enrolled 376 outpatients who underwent gastrointestinal endoscopy under midazolam sedation; 181 outpatients were assessed regarding discharge after sedative endoscopy using the MPADSS (group M), and 195 patients were assessed by the modified Aldrete score (group A). The clinical characteristics, types of endoscopy, endoscopic outcomes, and anesthesia outcomes were evaluated between the two groups. We compared discharge score, recovery time, and adverse events using propensity-score matching.

**Results:**

Propensity-score matching created 120 matched pairs. The proportion of patients who had a recovery time within 60 min after endoscopy was significantly higher in group A than that in group M (42.5% versus 25.0%, respectively; *P* < 0.01). The proportion of patients who required > 120 min of recovery time after endoscopy was significantly lower in group A than that in group M (0.0% versus 5.0%, respectively; *P* = 0.03). However, significantly more patients had drowsiness at discharge in group A compared with group M (19.1% versus 5.0%, respectively; *P* < 0.01). There was no significant difference in the adverse event rate within 24 h of discharge between the groups.

**Conclusions:**

Patients assessed by the modified Aldrete score were allowed to discharge earlier than those assessed by the MPADSS. However, a patient’s level of consciousness should be assessed carefully, especially in patients who visit the hospital alone.

**Supplementary Information:**

The online version contains supplementary material available at 10.1186/s12876-022-02549-7.

## Introduction

With developments in optical equipment, gastrointestinal endoscopy has become an indispensable technique for screening, diagnosing, and treating a variety of gastrointestinal diseases. However, because the procedure requires intubation with a flexible video endoscope, patients sometimes refuse the examination because of fear and anxiety [1, 2]. To improve patients’ acceptability, sedative endoscopy is being used increasingly [3, 4]. Sedation reduces patients’ fear and anxiety regarding endoscopy and also relieves the associated pain and discomfort [5–9]. These benefits can also be expected to lead to earlier detection of gastrointestinal pathologies, such as cancers [3, 10]. As a disadvantage, sedative endoscopy necessitates careful monitoring of a patient’s vital signs and consciousness during and after the procedure [11–13].

In Japan, intravenous sedation is usually performed by the endoscopists and/or nursing staff just before the endoscopy procedure [14–16]. The patients’ vital signs are monitored while they rest for a period of time in the recovery room after sedative endoscopy. Outpatients are allowed to discharge from the hospital when they are considered clinically stable, ready to go home, and able to rest at home [17, 18]. Thus, it is important to have clinically useful and objective discharge criteria to assess a patient’s condition after sedative endoscopy.

Several scoring systems have been proposed for the assessment of patients’ conditions following ambulatory surgery [19–21]. Among them, the modified post-anesthesia discharge scoring system (MPADSS) (see Additional Supplementary Table 1) has been used for the assessment of patients’ conditions following sedative colonoscopy [17, 22, 23]. The modified Aldrete score (see Additional Supplementary Table 2), originally developed to assess post-anesthetic recovery [24], has also been investigated for its usefulness for the assessment of a patient's condition at discharge after sedative endoscopy [25]. Because a clear discharge standard after sedative endoscopy has not been recommended in the recent guidelines [3], it is necessary to elucidate an appropriate discharge standard after sedative endoscopy.

In this study, we compared the patients’ clinical conditions and adverse events assessed by either the MPADSS or the modified Aldrete score, to determine the best discharge standard for outpatients after sedative endoscopy.

## Methods

### Patients and ethical issues

The present study enrolled outpatients who underwent sedative endoscopy at the National Hospital Organization Ureshino Medical Center from two prospective studies that analyzed the usefulness of MPADSS and the modified Aldrete score, respectively, as discharge standards. Outpatients > 20 years of age who underwent esophagogastroduodenoscopy, colonoscopy, or endoscopic ultrasonography under sedation were candidates for the present study. Intravenous sedation was not generally used in patients allergic to midazolam or in pregnant or breastfeeding women, thus excluding these patients. Outpatients who underwent therapeutic endoscopy were also excluded from the present study.

This study was conducted in accordance with the tenets of the Declaration of Helsinki and the guidelines of the Consolidated Standards of Reporting Trials (CONSORT). The study protocol and the consent procedure were approved by the Ethics Review Committee of the National Hospital Organization Ureshino Medical Center (approval number 19–03), and the study was registered with the University Hospital Medical Information Network (UMIN) Clinical Trials Registry (UMIN000037259) on 3 July 2019.

### Sedation and monitoring

Sedative and analgesic drugs were generally used as follows: An initial bolus of midazolam (3 mg for patients with a bodyweight of < 50 kg and 4 mg for patients with a bodyweight of ≥ 50 kg) was administered through an intravenous catheter. When the patients showed signs of discomfort, restlessness, agitation, and/or a response to verbal commands, 1 mg of midazolam was added as appropriate [5]. Analgesic agents (7.5 mg of pentazocine or 17.5 mg of pethidine hydrochloride) were also used if necessary. Analgesic drugs were selected by each endoscopist, considering the patient’s age or physical condition.

Vital signs, namely blood pressure, heart rate, and blood oxygen saturation were recorded before the induction of sedation. During the endoscopy, vital signs were monitored every 5 min. When oxygen saturation was < 92%, nasal oxygen supplementation (2 L/min) was administered. When a patient’s vital signs fluctuated by ≥ 20% compared with the baseline values, the endoscopic procedure was temporarily stopped until the values returned to baseline values.

Flumazenil, a benzodiazepine antagonist, was administered after the endoscopy as necessary.

### Assessment of the patients’ conditions after sedative endoscopy

Each patient’s clinical condition after sedative endoscopy was assessed by nurse-administered MPADSS as the discharge standard in 181 outpatients from July 2019 to January 2020 (group M). The patients’ conditions were assessed using a nurse-administered modified Aldrete score in 195 outpatients from July 2020 to December 2020 (group A). In both studies, the patients’ conditions were determined 60 min after the endoscopy. When the MPADSS or the modified Aldrete score reached ≥ 9 (maximum score: 10), patients were allowed to discharge from the hospital. Otherwise, the assessment was repeated every 30 min. When the patients did not reach a MPADSS or modified Aldrete score ≥ 9 3 h after endoscopy, the patient was admitted to the hospital.

### Data collection

The following patient demographic data were collected by reviewing the patients’ electronic clinical records: age, sex, alcohol consumption, smoking habit, body mass index, American Society of Anesthesiologists physical status (ASA-PS) classification, previous history of endoscopy, and comorbidities (including the Charlson comorbidity index score). Endoscopy-related data, namely the types of endoscopy (upper or lower gastrointestinal endoscopy, or endoscopic ultrasonography) and endoscopic outcomes (procedure time, types and amount of sedative, analgesic dose, antagonist, and endoscopist specialty), and peri-procedural adverse events (unstable vital signs indicated by ≥ 20% decrease from the baseline values) were collected by chart review.

After sedative endoscopy, the MPADSS or the modified Aldrete score at 60 min, recovery time, and vital signs before discharge were measured. Any adverse events before discharge and the need for hospitalization were recorded. Adverse events within 24 h after discharge were investigated by telephone questionnaire the day after endoscopy.

### Study endpoints

The primary endpoint of the present study was the difference in the discharge time after sedative endoscopy between the two groups. The secondary endpoint was the difference in the adverse events rate before discharge, need for hospitalization, and adverse events within 24 h after discharge between the two groups.

### Sample size calculation and statistical analysis

The sample size was estimated based on previously published results [17] and on the results from the previous data conducted from endoscopy in our hospital from July 2018 to June 2019. Based on these studies, we assumed that the rate of recovery time within 60 min after endoscopy to be 45%. We hypothesized that a difference of 20% in the rate of recovery time between the two groups would constitute a clinically meaningful difference. Assuming a power of 80% and an alpha of 0.025 (one-sided), at least 214 patients (107 patients in each group) would be required in this study. Assuming that 10% of patients could be lost to follow-up, a sample size of 236 patients (118 patients in each group) was planned.

**Fig. 1:**
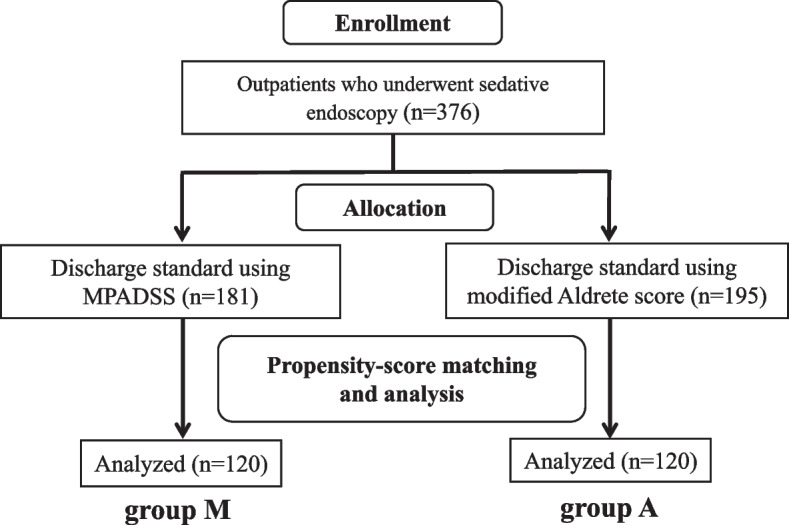
Study flow chart MPADSS: The modified post-anesthetic discharge scoring system, group M: discharge standard using the modified post-anesthetic discharge scoring system, group A: discharge standard using the modified Aldrete score

Propensity score matching analysis was used to assess the clinical usefulness of MPADSS and the modified Aldrete score as the discharge criteria. This method was used to adjust for significant differences in the patients’ baseline characteristics and to minimize the influence of possible confounding factors [18, 26]. The two groups were matched at a 1:1 ratio (120 patients in each group) with adjustment for seven covariates (age, sex, ASA-PS classification, comorbid malignant diseases, procedure time, mean midazolam dose, use of analgesics) to minimize inherent bias (Fig. 1). These seven covariates were selected based on the opinions of expert endoscopists (DY, TM, NH, YT, and ST). This model yielded a C statistic of 0.754, indicating a preferable ability for the comparison between groups M and A. The caliper width of the propensity score matching was 0.20.

Categorical data were expressed as frequency and percentage, and the chi-square test was used to identify differences between the two groups. Normally-distributed numerical data were expressed as mean ± standard deviation, and Student’s *t*-test was used to determine differences between the two groups. Numerical data with a skewed distribution were expressed as median [interquartile range], and the Mann–Whitney *U*-test was used for comparisons between the groups. Levels of significance for all comparisons were reported, regardless of statistical significance, as *P* values or confidence intervals. A *P* value < 0.05 was considered statistically significant for each test. All statistical analyses were performed with JMP, version 13.2.0 (SAS Institute Inc., Cary, NC, USA).

## Results

### Characteristics of patients and the propensity-score matched pairs

The present study included 376 outpatients from the two prospective studies. The patients’ baseline characteristics and endoscopic outcomes are listed in Table 1. When comparing the baseline characteristics between the two groups, sex, ASA-PS classification, and comorbid malignant disease were significantly different between groups M and A. While no significant difference in the type of endoscopy was found between the two groups, endoscopic outcomes were significantly different regarding procedure times, mean doses of midazolam, and the use of an analgesic agent. The rate of adverse events did not differ between the two groups.

**Table 1:** Characteristics of the patients and endoscopy outcomes

	Group M (%)	Group A (%)	*P* value
Number of patients (N)	181	195	
Age (years)	64.2 ± 14.3	62.8 ± 15.7	0.38
Sex, male	53 (29.3%)	85 (43.6%)	< 0.01
Drinking	59 (32.6%)	81 (41.5%)	0.09
Smoking	39 (21.6%)	59 (30.3%)	0.06
BMI (kg/m2)	22.4 ± 3.6	22.2 ± 3.9	0.69
ASA-PS classification, I	170 (93.9%)	192 (98.5%)	0.03
Previous history of endoscopy	135(74.6%)	130 (66.7%)	0.11
Comorbidity			
Cardiovascular diseases	24 (13.3%)	26 (13.3%)	1.00
Cerebrovascular diseases	9 (5.0%)	7 (3.6%)	0.61
Chronic kidney diseases	5 (2.8%)	6 (3.1%)	1.00
Chronic liver damage	4 (2.2%)	6 (3.1%)	0.75
Diabetes mellitus	34 (18.8%)	27 (13.9%)	0.21
Hypertension	63 (34.8%)	76 (39.0%)	0.45
Malignant diseases	54 (29.8%)	83 (42.6%)	0.01
Charlson comorbidity index	1.5 ± 1.6	1.3 ± 1.3	0.11
Types of endoscopy			
Upper Gastrointestinal	117 (64.6%)	134 (68.7%)	0.44
Lower Gastrointestinal	59 (32.6%)	48 (24.6%)	0.11
Endoscopic ultrasonography	5 (2.8%)	12 (6.2%)	0.14
Endoscopic outcomes			
Procedure time (min)	15.8 ± 7.7	20.0 ± 11.6	< 0.01
Mean midazolam dose (mg)	4.7 ± 1.6	4.0 ± 1.5	< 0.01
Using analgesic agent	57 (31.5%)	37 (19.0%)	0.01
Mean pentazocine dose (mg)	10.1 ± 3.6	9.0 ± 3.1	0.18
Mean pethidine dose (mg)	34.2 ± 3.7	35.0 ± 0.0	0.33
Using antagonist	3 (1.7%)	0 (0.0%)	0.11
Operator of trainee	89 (49.2%)	110 (56.4%)	0.18
Adverse events	2 (1.1%)	1 (0.5%)	0.61
Desaturation	1 (0.6%)	1 (0.5%)	1.00
Hypotension	1 (0.6%)	0 (0.0%)	1.00

Results are presented as the number of patients or mean ± standard deviation. *BMI* Body mass index, *ASA-PS* American Society of Anesthesiologists physical status, group M: discharge standard using the modified post-anesthetic discharge scoring system, group A: discharge standard using the modified Aldrete score

Propensity-score matching created 120 matched pairs. After propensity score matching, no difference was found regarding the seven selected covariates between group M and group A (Table 2).

**Table 2:** Characteristics before and after propensity-score matching in groups M and A

Before propensity-score matching				
	group M (%)	group A (%)	*P* value	Standardized difference
Number of patients (N)	181	195		
Age (years)	64.2 ± 14.3	62.8 ± 15.7	0.38	0.09
Sex, male	53 (29.3%)	85 (43.6%)	< 0.01	0.30
ASA-PS classification, I	170 (93.9%)	192 (98.5%)	0.03	0.24
Malignant diseases	54 (29.8%)	83 (42.6%)	0.01	0.27
Procedure time (min)	15.8 ± 7.7	20.0 ± 11.6	< 0.01	0.43
Mean midazolam dose (mg)	4.7 ± 1.6	4.0 ± 1.5	< 0.01	0.45
Using analgesic agent	57 (31.5%)	37 (19.0%)	0.01	0.29
After propensity-score matching				
	group M (%)	group A (%)	*P* value	Standardized difference
Number of patients (N)	120	120		
Age (years)	63.4 ± 15.2	62.7 ± 15.6	0.76	0.05
Sex, male	42 (35.0%)	35 (29.2%)	0.41	0.12
ASA-PS classification, I	117 (97.5%)	117 (97.5%)	1.00	0.00
Malignant diseases	43 (35.8%)	39 (32.5%)	0.68	0.07
Procedure time (min)	17.0 ± 8.0	16.8 ± 8.6	0.81	0.02
Mean midazolam dose (mg)	4.4 ± 1.6	4.3 ± 1.7	0.67	0.06
Using analgesic agent	29 (24.2%)	23 (19.2%)	0.43	0.12

Results are presented as the number of patients or mean ± standard deviation. ASA-PS: American Society of Anesthesiologists physical status, group M: discharge standard using the modified post-anesthetic discharge scoring system, group A: discharge standard using the modified Aldrete score

### Study endpoints

Table 3 shows the results of the comparisons of discharge scores, recovery times, and adverse events before discharge between the two groups. Overall, the MPADSS score 60 min after sedative endoscopy was 9.4 ± 0.9, and the modified Aldrete score was 9.3 ± 1.1.

**Table 3:** Discharge score, recovery time, and adverse events before discharge in groups M and A

	group M (%)	group A (%)	*P* value
Number of patients (N)	120	120	
MPADSS or Modified Aldrete score at 60 min	9.4 ± 0.9	9.3 ± 1.1	0.38
Recovery time (min)*	60 (60–82.5)	60 (60–86.3)	0.91
Recovery time ≦ 60 (min)	30 (25.0%)	51 (42.5%)	< 0.01
Recovery time ≧ 120 (min)	6 (5.0%)	0 (0.0%)	0.03
Vital signs at discharge			
Mean systolic pressure (mmHg)	122.5 ± 18.5	121.8 ± 20.4	0.78
Mean diastolic pressure (mmHg)	73.0 ± 13.1	70.8 ± 12.4	0.19
Mean pulse (/min)	67.4 ± 11.0	66.4 ± 11.2	0.48
Mean oxygen saturation (%)	97.6 ± 1.7	97.5 ± 2.0	0.70
Adverse events at discharge			
Drowsiness	6 (5.0%)	23 (19.1%)	< 0.01
Bad feeling	3 (2.5%)	4 (3.3%)	1.00
Nausea	0 (0%)	2 (1.7%)	0.50
Hospitalization	1 (0.8%)	0 (0%)	1.00

Results are presented as the number of patients or mean ± standard deviation. *Values are given as median [interquartile range]. MPADSS: the modified post-anesthetic discharge scoring system, group M: discharge standard using the modified post-anesthetic discharge scoring system, group A: discharge standard using the modified Aldrete score

The proportion of patients who had a recovery time within 60 min after endoscopy was significantly higher in group A than that in group M (42.5% versus 25.0%, respectively; *P* < 0.01). The proportion of patients who had a recovery time > 120 min after endoscopy was significantly lower in group A than that in group M (0.0% versus 5.0%, respectively; *P* = 0.03). However, the mean recovery time was comparable between the two groups. The patients’ vital signs before discharge were not significantly different between the two groups.

Drowsiness was seen more frequently in patients in group A compared with group M as an adverse event before discharge (5.0% versus 19.1%, respectively;* P* < 0.01), whereas no other adverse event rates differed between the groups. In group M, one patient required hospitalization because of inadequate arousal after sedative endoscopy.

Table 4 shows the comparison of the adverse events within 24 h of hospital discharge between the two groups. While drowsiness was the most frequent adverse event within 24 h in both groups, the rates of adverse events within 24 h of discharge were not significantly different between the two groups. Most of the adverse symptoms were mild and resolved within 24 h. However, three patients in group M and two patients in group A returned to the hospital within 24 h of discharge for further observation. One of these patients was admitted to the hospital at the patient’s request.

**Table 4:** Adverse events within 24h of hospital discharge in groups M and A

	group M (%)	group A (%)	*P* value
Number of patients (N)	120	120	
Adverse events within 24h			
Drowsiness	38 (31.7%)	43 (35.8%)	0.59
Bad feeling	10 (8.3%)	11 (9.2%)	1.00
Nausea	9 (7.5%)	6 (5.0%)	0.60
Vomiting	6 (5.0%)	4 (3.3%)	0.75
Abdominal pain	5 (4.2%)	3 (2.5%)	0.72
Abdominal fullness	5 (4.2%)	7 (5.8%)	0.77
Fever	2 (1.7%)	2 (1.7%)	1.00
Return to the hospital	3 (2.5%)	2 (1.7%)	1.00

Group M: discharge standard using the modified post-anesthetic discharge scoring system, group A: discharge standard using the modified Aldrete score

## Discussion

Because the use of sedation can improve the acceptability of gastrointestinal endoscopy, sedative endoscopy has been introduced in many hospitals in Japan [14–16]. Guidelines for sedation in gastroenterological endoscopy have been provided [3]. However, the increasing number of sedative endoscopy procedures has highlighted the requirement for space and personnel for patient recovery. Furthermore, this increase also highlighted the lack of simple and objective criteria to assess patients’ physical conditions for safe discharge from the hospital. Discharge of patients after sedative endoscopy is generally determined in accordance with the comprehensive discretion of the physicians. Cognitive and psychomotor tests are used for the assessment in some institutions; however, these tests are labor-intensive and time-consuming [27–29]. The clinical usefulness of the observer's assessment of alertness/sedation scale and the Ramsay sedation score for the assessment of sedation levels have been reported previously [30, 31]. However, the validity of these systems for discharge assessment remains uncertain. In the present study, we evaluated the clinical impact of the MPADSS and the modified Aldrete score after sedative endoscopy, considering their suitability as discharge standards after sedative endoscopy. We used these systems because they are widely accepted discharge standards after ambulatory surgery [20, 21, 23–25, 30–33].

In the present study, the subjects were enrolled from the two prospective studies that analyzed the clinical usefulness of the nurse-administered MPADSS or the modified Aldrete score as the discharge standard after sedative endoscopy. Therefore, a significant difference in the patients’ demographics and endoscopy-related factors were found between groups M and A. Thus, we used propensity score matching analysis to adjust for the significant differences in the patients’ baseline characteristics and to minimizes the influence of possible confounding factors.

After propensity score matching, although the mean recovery time after sedative endoscopy did not differ between the two groups, more patients in group A were judged to reach the conditions for discharge at 60 min compared with group M. In addition, six patients in group M required more than 2 h to judge that they were safe to discharge. Thus, it can be considered that using the modified Aldrete score shortens the recovery time and uses the endoscopy recovery room more effectively.

In contrast, when comparing adverse events between the two groups, more patients felt drowsiness at discharge in group A compared with group M. This higher rate of drowsiness in group A than that in group M appears to be attributed to the higher number of patients judged safe for discharge within 60 min in group A compared with group M. The difference in the assessment of consciousness level between the two groups could also have contributed to the results. This is because patients judged as arousable upon calling for the consciousness level assessment in the modified Aldrete score can be allowed to discharge if the scores for the remaining assessments are perfect. While the rates of patients who required further observation after discharge were not significantly different between the two groups, a patient’s level of consciousness should be assessed carefully using the modified Aldrete score, especially in patients who visit the hospital alone for sedative endoscopy.

Most of the adverse events within 24 h after discharge in both groups in this study were mild and resolved within 24 h. In this sense, both the MPADSS and the modified Aldrete score can be considered to meet the needs for discharge standards after sedative endoscopy. However, three patients in group M and two patients in group A returned to the hospital within 24 h of discharge for further observation. Therefore, patients and their families should be cautioned regarding possible continuation or deterioration of adverse events even after discharge.

Our study has several limitations. First, this was a single-institution study. Second, this study was not a randomized trial. Well-designed prospective studies are necessary to validate the results of the present study. Third, while the MPADSS and the modified Aldrete score are both simple to use, these systems cannot evaluate all adverse events, such as hypoglycemia. Additionally, the MPADSS includes the item, surgical bleeding, which is inadequate for the assessment of a patient’s condition after sedative endoscopy. No case was defined as positive for hematemesis or bloody stool in the present study.

In conclusion, the modified Aldrete score can be considered more appropriate than the MPADSS as the discharge standard after sedative endoscopy from the viewpoints of the assessment items of the scores and the effective use of the endoscopy recovery room. However, a patient’s level of consciousness should be assessed carefully when using the modified Aldrete score, especially in patients who visit the hospital alone.

## Supplementary Information

**Additional file 1: Supplementary Table 1.** The modified post-anesthetic discharge scoring system.** Supplementary Table 2.** The modified Aldrete score.

## Data Availability

The datasets used and/or analyzed during the current study are available from the corresponding author on reasonable request.

## Abbreviations

MPADSS: Modified post-anesthesia discharge scoring system
ASA-PS: American Society of Anesthesiologists physical status
