# Supplementary material for: Usefulness of discharge standards in outpatients undergoing sedative endoscopy: a propensity score-matched study of the modified post-anesthetic discharge scoring system and the modified Aldrete score
Source: BMC Gastroenterol. 2022 Nov 4;22:445. doi: 10.1186/s12876-022-02549-7 (PMC9635164; doi:10.1186/s12876-022-02549-7)
Supplement: Supplementary file 1 — Additional file 1: Supplementary Table 1. The modified post-anesthetic discharge scoring system. Supplementary Table 2. The modified Aldrete score. [file 12876_2022_2549_MOESM1_ESM.docx]

**Supplementary Table 1** The modified post-anesthetic discharge scoring system

| Categories | Points |
| --- | --- |
| Vital signs |  |
| BP and HR ± 20% of pre-endoscopy value | 2 |
| BP and HR ± 20%-40% of pre-endoscopy value | 1 |
| BP and HR ± 40% of pre-endoscopy value | 0 |
| Activity |  |
| Steady gait, no dizziness or meets pre-endoscopy level | 2 |
| Requires assistance | 1 |
| Unable to ambulate | 0 |
| Nausea and vomiting |  |
| No or minimal/treated with p.o. medication | 2 |
| Moderate/treated with parenteral medication | 1 |
| Severe/continues despite treatment | 0 |
| Pain |  |
| Minimal or no pain (Numerical Analogue Scale = 0-3) | 2 |
| Moderate (Numerical Analogue Scale = 4-6) | 1 |
| Severe (Numerical Analogue Scale = 7-10) | 0 |
| Surgical bleeding |  |
| None or Minimal (not requiring intervention) | 2 |
| Moderate (1 episode of hematemesis or rectal bleeding) | 1 |
| Severe (≥ 2 episodes of hematemesis or rectal bleeding) | 0 |

Discharge standards are satisfied if the score is ≥ 9.

BP: blood pressure, HR: heart rate, p.o.: per os

**Supplementary Table 2** The modified Aldrete score

| Categories | Points |
| --- | --- |
| Respiration |  |
| Able to breathe deeply and cough | 2 |
| Dyspnea or shallow breathing Apnea | 1 |
| Apnea | 0 |
| Oxygen saturation (SpO2) |  |
| Maintains > 92% on room air | 2 |
| Needs O2 inhalation to maintain O2 saturation ≧ 90% | 1 |
| SpO2 < 90% (with supplemental oxygen administered) | 0 |
| Consciousness level |  |
| Fully awake | 2 |
| Arousable upon calling | 1 |
| Not responding | 0 |
| Circulation |  |
| BP ± 20 mmHg (relative to pre-operation standard value) | 2 |
| BP ± 20–50 mmHg (relative to the pre-procedural standard value) | 1 |
| BP ± 50 mmHg (relative to the pre-procedural standard value) | 0 |
| Activity |  |
| Able to move the four extremities | 2 |
| Able to move two extremities | 1 |
| Cannot move the four extremities | 0 |

Discharge standards are satisfied if the score is ≥ 9.

BP: blood pressure
